# EXPLORING THE UNIQUE NEEDS AND EXPERIENCES OF BLACK MALE CAREGIVERS

**DOI:** 10.1093/geroni/igae098.0125

**Published:** 2024-12-31

**Authors:** Robert Turner

**Affiliations:** The George Washington University School of Medicine and Health Sciences, Washington, District of Columbia, United States

## Abstract

An emerging area of public health concern for policymakers, caregivers and families, and health service workers is the growing number of older adults with Alzheimer’s disease and related dementia (ADRD). The number of older Black Americans with ADRD who will require some care is projected to triple in the upcoming decade. According to survey data, 30% to 40% of informal caregivers are elderly husbands often caring for spouses experiencing dementia; however, this estimation may be low simply because many men – especially Black American men – do not formally identify as caregivers. Previous research also suggests that Black American men often feel unprepared as caregivers, are disinclined to discuss this role, and rely on informal support resources due to hesitation or unawareness. Although Black Americans are at greater risk of caregiver burden (CB), the body of research that focuses on adult Black American men as primary caregivers of a person with dementia (PWD) is sparse. Knowledge about dementia CB in this understudied sub-population is critical for several reasons, including the rapid projected growth that puts their health and well-being at risk. The Black Male Dementia Caregiver Burden Study examines the impact of dementia CB on the cognitive function and physiological health of this underrepresented population. Additionally, this project incorporates focus group and in-depth interview data that explores Black males’ unique needs and experiences with caregiving while addressing their concerns about participating in caregiver research, including collecting biospecimen samples (e.g., urine, blood, saliva).

